# Glucose-Regulated Protein 94 Mediates the Proliferation and Metastasis through the Regulation of ETV1 and MAPK Pathway in Colorectal Cancer

**DOI:** 10.7150/ijms.56024

**Published:** 2021-03-27

**Authors:** Uyanga Batzorig, Po-Li Wei, Weu Wang, Chien-Yu Huang, Yu-Jia Chang

**Affiliations:** 1 International Ph.D. Program in Medicine, College of Medicine, Taipei Medical University, Taipei 110, Taiwan.; 2 Division of Colorectal Surgery, Department of Surgery, Taipei Medical University Hospital, Taipei Medical University, Taipei 110, Taiwan.; 3 Department of Surgery, School of Medicine, College of Medicine, Taipei Medical University, Taipei 110, Taiwan.; 4 Cancer Research Center, Taipei Medical University Hospital, Taipei Medical University, Taipei 110, Taiwan.; 5 Graduate Institute of Cancer Biology and Drug Discovery, Taipei Medical University, Taipei 110, Taiwan.; 6 Division of General Surgery, Department of Surgery, Shuang Ho Hospital, Taipei Medical University, Taipei, Taiwan.; 7 Division of Colorectal Surgery, Department of Surgery, Shuang Ho Hospital, Taipei Medical University.; 8 Graduate Institute of Clinical Medicine, College of Medicine, Taipei Medical University, Taipei 110, Taiwan.; 9 Department of Pathology, Wan Fang Hospital, Taipei Medical University, Taipei, Taiwan.; 10 Cell Physiology and Molecular Image Research Center, Wan Fang Hospital, Taipei Medical University, Taipei, Taiwan.

**Keywords:** GRP94, colorectal cancer, ETV1, proliferation, metastasis.

## Abstract

Colorectal cancer (CRC) is a worldwide health problem. Glucose-regulated protein 94 (GRP94) is known as an important endoplasmic reticulum-stress response protein that shows correlation with aggressive cancer behavior. However, the role of GRP94 in CRC is still unclear. Our results showed that silencing GRP94 (GRP94-KD) reduced cell proliferation, invasion and migration of CRC cells and suppressed tumorigenesis in the xenograft mouse model. Rescue assay showed that ETV1 overexpression reversed the effect of GRP94 on cell proliferation and migration. In the molecular mechanism, we found that knockdown of GRP94 inhibited the level of MAPK pathway, including ERK/p-ERK, JNK/p-JNK, and p38/p-p38 signals. Cyclooxygenase-2 and epithelial-mesenchymal transformation biomarkers, such as N-cadherin, vimentin, and β-catenin were suppressed in GRP94 knockdown cells. Treatment of specific inhibitors of MAPK pathway showed that ERK/p-ERK, and p38/p-p38 inhibitors significantly influenced ETV1 expression as compared to JNK/p-JNK inhibitor. Our results indicated that silencing GRP94 repressed the ability of EMT process, cancer cell proliferation, metastasis, and CRC tumorigenesis. Therefore, GRP94 may play an important role in CRC by regulating ETV1 and MAPK pathway.

## Introduction

Colorectal cancer (CRC) is one of the most common malignant cancers with more than one million new cases worldwide, causing 600000 deaths annually 1. The recurrence rate in advanced-stage of CRC patients was over 30% 2. Standard treatments for patients with metastatic CRC are chemotherapy, radiotherapy and new-generation biological agents 3. The resistance to traditional drug, 5-FU, was approximately 50% for aggressive cancer, and 5-year survival rate of patients with metastatic CRC was around 12% 3, 4. Therefore, finding a target gene is important to understand the pathogenesis of CRC.

The glucose-regulated protein 94 (GRP94) is the heat shock protein encoded by *HSP90B1* in human and is first noted after heat induction 5, 6. GRPs are located in the cytosol, nucleus, and organelles that play important roles in metabolic balance and regulation of protein quality control 5. Most of the GRPs, located on the endoplasmic reticulum (ER), function as facilitating protein folding, congregation and sending misfolded proteins for degradation 7. Induction of GRPs is required for cancer progression by helping cancer cells to survive by escaping immune system, working against apoptosis, and increasing resistance to chemotherapy 8. The higher response of GRPs to endoplasmic reticulum stress was more related to aggressive and resistant cancer behavior 9, 10. Higher level of GRP94 expression was associated with poorly differentiated phenotype and metastasis of colon cancer 11. In contrast, Ryan et al., reported that the expression of GRP94 was not correlated with disease stages of CRC 12. Therefore, the role of GRP94 in CRC is still unclear and needs to be studied. To study the underlying mechanism of GRP94 in CRC, we used nanostrip to find the downstream target of GRP94 and result showed that ETV1 was the best candidate.

ETV1, ETS (E26 transformation-specific family) translocation variant, or ETS-related protein 81 (ER81) belongs to ETS transcription factor family and is phosphorylated by mitogen-activated protein kinase (MAPK) 13. ETV1 is an oncogenic transcription factor of PEA3 subfamily of ETS transcription factors characterized by a winged helix-turn-helix DNA-binding motif 14, 15. ETV1 is a target of the MEK/ERK/Ras/Raf signaling pathway and activated ERK-1 is capable of phosphorylating ETV1 16. GRP94 is a member of HSP90 family and HSP90 inhibitors suppressed the HER2 positive triple negative breast cancers through downregulation of the Ras/Raf/MAPK pathway 17. Previous studies showed that silencing GRP94 decreased the expression level of protein of MAPK signaling in HCC and esophageal squamous cell carcinoma 18, 19. ETV1 phosphorylated by downstream MAPK signaling increased protein stability and instability of ETV1 inhibited the growth ability of gastrointestinal stromal tumor cells 20 21. YK-4-279, inhibitor of ETV1, suppressed primary tumor development and metastasis in fusion positive mice with prostate cancer 22. In three-dimensional organoid models of pancreatic ductal adenocarcinoma, up-regulation of ETV1 disrupted the cyst architecture and then increased invasive capacity of pancreatic cancer by inducing EMT 23. ETV1 induced EMT, metastasis and stromal expansion through part of the Sparc and Has2 in mice model of pancreatic cancer 23. ETV1 increases the EMT in human gastric cancer cells by raising expression of SNAIL 24. 47% of 100 colorectal cancer tissues had high expression of ER81 (ETV1), however, its expression level was not correlated with the clinicopathological findings and expression of MMPs 25. Genetic screening findings from 39 patients with colorectal cancer showed ETV1 was significantly correlated with the lymphatic metastasis of colorectal cancer 26. However, another study reported that ETV1 expression was not related to the clinicopathological findings 25. Therefore, based on the aforementioned studies, we hypothesized that GRP94 may regulate cancer progression through ETV1 in CRC.

This study presents that silencing GRP94 may suppress the ability of proliferation, metastasis and invasion of CRC cells through downregulation of ETV1 expression, and the possible signaling pathways.

## Materials and Methods

### Cell culture and chemicals

The CRC cell lines, HCT 116, and DLD-1, were purchased from American Type Culture Collection (ATCC) (Manassas, VA, USA). The RPMI-1640 containing 10 % FBS (fetal bovine serum) (Gibco life technologies) and 1% penicillin-streptomycin (10,000 U/mL penicillin and 10 mg/mL streptomycin) were used to culture the cells in 37^0^C humidified incubator with 5% CO_2_. Anti-GAPDH, anti-β-catenin, anti-vimentin, and anti-GRP94 antibodies were purchased from Santa Cruz Biotechnology (Santa Cruz, CA, USA). Anti-p38, anti-phospho-p38, anti-E-cadherin, anti-N-cadherin, anti-JNK, anti-phospho-JNK, anti-ERK, anti-phospho-ERK, and anti-COX-2 antibodies were purchased from Cell Signaling Technology (Danvers, MA, USA). Anti-ETV1 antibody was obtained from GeneTex (Alton Pkwy, USA) and Abcam (Cambridge, MA, USA). SB203580(P38 inhibitor), PD98059(MEK inhibitor), and SP600125(JNK inhibitor) inhibitors (Selleck Chemicals, UK) were added into the cells and incubated for 2, 4, and 8 hours at 37°C in 5% CO_2_ in a humidified incubator. Then, cell pellets were collected for western blot.

### Silencing GRP94 expression by shRNA

The GRP94 specific shRNA and shRNA control were obtained from the National RNAi Core Facility, Academia Sinica, Taiwan, as described previously 27. Small hairpin RNA (shRNA) was used to knockdown GRP94 in CRC cells. The non-target control shRNA vector (pLKO.1<-puro) and GRP94-shRNA plasmids were transfected into cells by Neon Transfection system (Invitrogen Life Technologies, Grand Island, NY), and stably transfected cells were selected by using puromycin as previously reported 28.

### Western Blot Analysis

Following SB203580(P38 inhibitor, 3μM), PD98059(MEK inhibitor, 50μM), and SP600125(JNK inhibitor, 50μM) inhibitors treatment for 8 hours, cell pellets were collected and were lysed by lysis buffer containing M-PER reagent, phosphatase and protease inhibitor (Boehringer Mannheim, Indianapolis, IN). 20µg total protein was separated on 10% SDS (sodium dodecyl sulphate)-polyacrylamide gel and transferred into a polyvinylidene fluoride membrane (Pall Corp, Port Washington, NY, USA). Membranes were blocked with 5% milk at room temperature for 1 hour. The primary antibodies (anti-GPR94(SC-1794), anti-β-catenin (SC-7963), anti-vimentin (SC-6260), anti-GAPDH(SC-32233) anti-E-cadherin (3195S), anti-N-cadherin (13116S), anti-p38 (9212S), anti-phospho-p38 (9211S), anti-JNK(9252S), anti-phospho-JNK (4668S), anti-ERK (9102S), anti-phospho-ERK (4370S), anti-ETV1(GTX129202) (ab81086) and anti-COX-2(SC-19999)) were incubated at 4°C for overnight. Membranes were washed three times with 1xPBS. Primary antibody reactivity was detected by using horseradish-peroxidase-conjugated donkey anti-rabbit, anti-mouse, and anti-goat secondary antibodies (Santa Cruz Biotechnology, Dallas TX, USA) at room temperature for 2 hours and visualized by using the Super Signal West Pico Chemiluminescent Substrate (GE, Healthcare, Piscataway, NJ) by Versa Doc 5000 Imaging system (Bio-Rad Laboratories) 28, 29.

### RNA collection, cDNA synthesis, and real-time PCR analysis

Total RNA was extracted from fresh-frozen colorectal cancer cell lines (scrambled control and GRP94-KD cells) by using RNAzol®RT according to protocol provided by the manufacturer (Molecular Research Center, Inc., Ohio). 8μg RNA was reverse transcribed into cDNA by using High-Capacity cDNA Reverse Transcription Kit and then expanded by Applied Biosystems® 7500 Real-Time PCR Systems (Applied BioSystem, US) with the QPCR kit. Polymerase chain reaction (PCR) amplifications of GRP94 and ETV1 were carried out with 5µl SYBRGreen (Bio-Rad Laboratories, Hercules, CA, USA), 0.5µl complementary DNA, 3.5µl ddH_2_0, 1µl forward and reverse primers (GRP94 (10µM), ETV1(10µM) and GAPDH (10µM)) in a final volume of 10 µl 28.

### Cell viability assay

Proliferative ability of the CRC cells was evaluated by SRB assay. 5000 cells from scrambled control and GRP94-KD cells were seeded in each well of 96-well micro plates and incubated for 0-3 days at 37^0^ C. After 24, 48, and 72 hours, medium was removed and TCA was added to fix the cells. The supernatant was removed and 0.4% (w/v) SRB solution (prepared in 1% acetic acid) was added to the wells. Cells were incubated with SRB solution for 20 min at room temperature. 1% acetic acid was used to remove unbound dye and then 150 µl tris base (20mM, pH=7.4) was added into each well to elute the bound stain. After 30 minutes incubation at shaker, the absorbance was determined at 515nm 30.

### Cell counting kit-8 (CCK-8) assay

Cell survival rates were evaluated by CCK-8 assay (Dojindo Laboratories, Kumamoto, Japan). 15x10^3^ cells were seeded in each well of 96-well plates and treated with 3μM P38 inhibitor (SB203580), 50μM MEK inhibitor (PD98059), and 50μM JNK inhibitor (SP600125) for 8 hours. 100μl CCK-8 solution was added to each well and absorbance was determined at 450nm after 2 hour incubation at 37 °C.

### Transwell migration assay

Migration ability was determined by using a BD Falcon cell culture insert (BD Biosciences, San Jose, CA). The cell culture insert was placed in each well containing 1ml culture medium and then 1x10^5^ cells suspended in 500µl serum-free medium were seeded into the culture insert. After 48h incubation at 37°C in 5% CO_2_, each well and insert were washed once with 1ml 1xPBS. The cells were fixed with 1ml methyl alcohol solution for 1 minute. The cells which did not migrate were removed from the upper surface of the membrane. The migrated cells were stained with 0.1% crystal violet. After incubating the plate for 2 hours at room temperature, crystal violet was removed and stained cells were washed with 1xPBS. Then, cells were counted under a microscope (Olympus IX) at 10X. The migrated cells were counted by hand cell counter and Image J software.

### Invasion assay

BD BioCoat Matrigel™ invasion chambers (BD Biosciences, USA) pre-coated with BD Matrigel matrix were used according to the protocol provided by a manufacturer. The invasion chamber was placed in each well containing 1ml of culture medium and then 1x10^5^ cells suspended in 500µl serum-free medium were seeded into the chamber pre-coated with matrigel. After incubation for 48 hours at 37°C in 5% CO_2_, each well and cup were washed once with 1ml 1xPBS. The procedures for fixation, staining, and counting were same as “Transwell migration”.

### Establishment of *in-vivo* tumor xenograft experiments

DLD-1 cells that possessed stable integrations of GRP94-shRNA and scrambled control sequences were established. 5 weeks old male Nu/Nu mice were used as *in-vivo* experimental model as described previously 31, 32. 10^7^cells/ml of GRP94-KD DLD-1 and scrambled control cells were suspended in PBS. 0.2ml cell suspension was injected subcutaneously (s.c.) in the flank of mouse. Total 16 mice (8 mice for each group) were used for one set experiment. The body weight and tumor dimensions were measured twice per week. We measured subcutaneous tumors with the equation (L × w^2^)/2 in accordance with the rules of Taipei Medical University Animal Care and Use. After sacrificing the mice, tumors were excised and weighed. The xenografts were either flash frozen on dry ice and kept at -80°C for analyzing RNA and protein; or fixed in 10% formalin and then embedded in paraffin for IHC (immunohistochemical staining) staining. All the protocols for animals were approved by the Institutional Animal Care and Use Committee (IACUC) of Taipei Medical University (LAC-2014-0401).

### Plasmid rescue assay

ETV1 plasmid was purchased from Origene (OriGene Technologies, Inc). DNA was extracted by plasmid miniprep purification kit (GeneMark, Taiwan). Plasmid rescue assay was performed by using electroporation by Neon®Transfection System (Invitrogen Life Technologies, Grand Island, NY) in accordance with the manufacturer's protocol. Briefly, 2x10^6^ cells were harvested and mixed with 1µl ETV1 plasmid DNA and 9µl T-buffer and loaded into the Neon Pipette tip. Transfection was performed at 1000 voltage with 20ms width. After 48 hours, cells were collected and mRNA expression of ETV1 was measured by QPCR.

### Statistical analysis

All experiments were repeated at least three times. All of the data were presented as the mean ± standard deviation (SD). Statistical significance was determined using unpaired student's t test (two-tailed) or two-way ANOVA to compare two datasets by Prism 8 software (Graph-Pad Software, In., San-Diego, CA). A value of p < 0.05 was considered statistically significant.

## Results

### GRP94 mediates the cell growth activity in CRC cells

**Figure 1:**
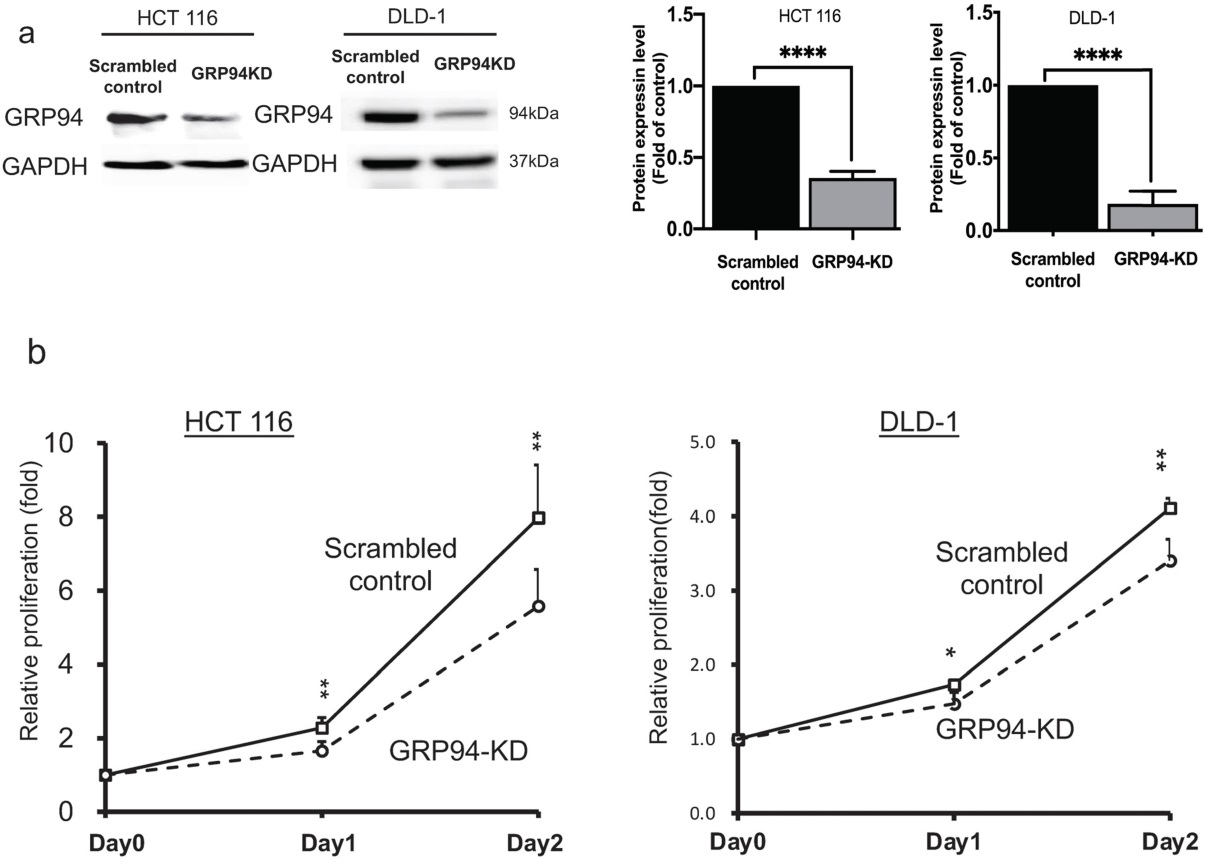

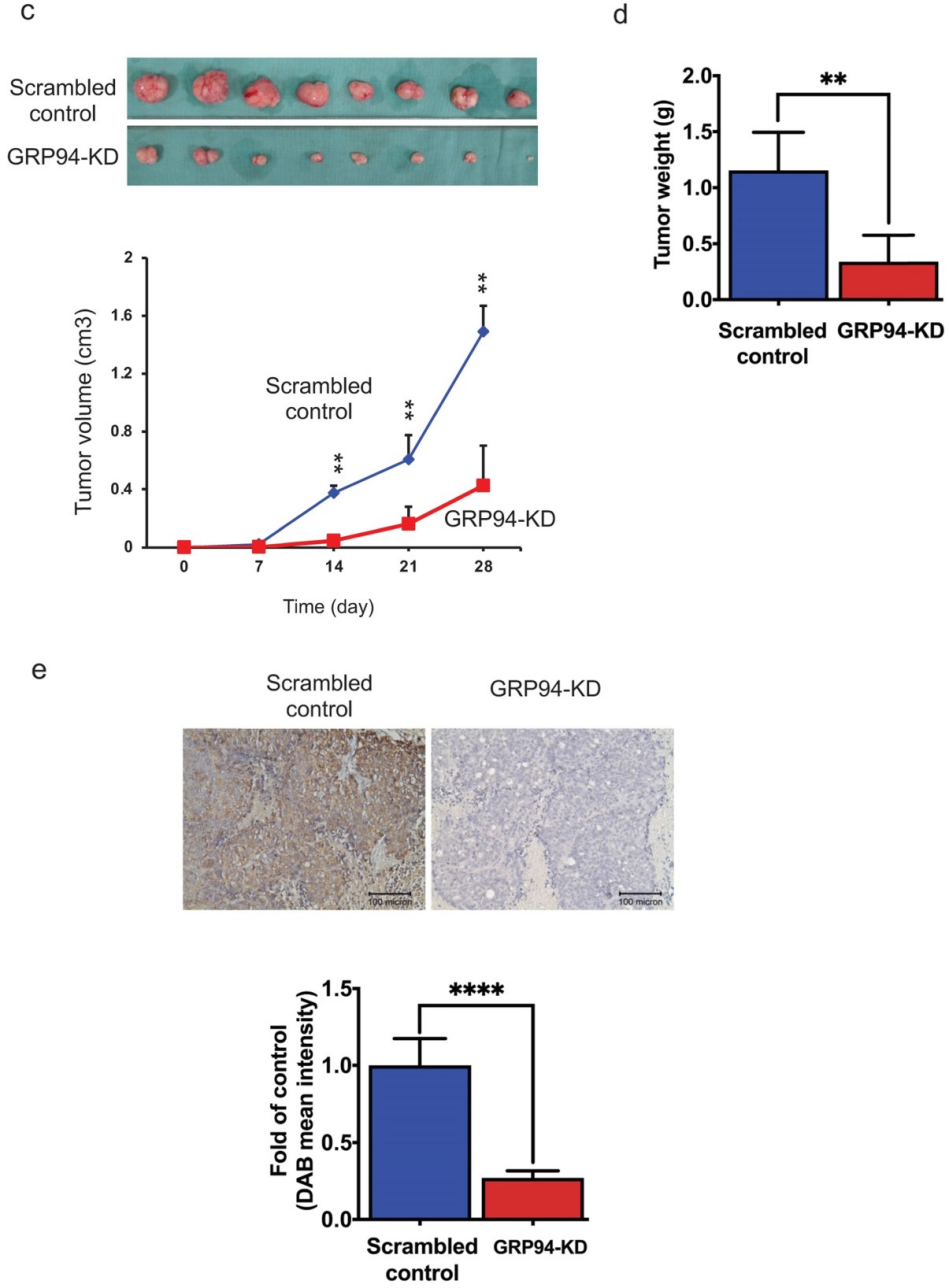
** GRP94 mediates cancer progression *in vitro* and *in vivo*.** (a) Expression level of GRP94 in GRP94-KD and scrambled control cells was evaluated by Western blotting (n=3). Quantitative data was normalized to GAPDH. (b) Cell proliferation of scrambled control and GRP94-KD cells was determined by SRB assay (n=3). (c) In xenograft model, volume of GRP94-KD tumor was smaller than scrambled control tumor (8 mice for each group). (d)Tumor weight of GRP94-KD was less than that of scrambled control. (e) Immunohistochemistry staining (IHC) showed negative reaction in GRP94-KD group and positive reaction in scrambled control group. (*P<0.05, **P <0.01, ****P < 0.0001)

To evaluate the role of GRP94 in cell proliferation of CRC, we generated GRP94-KD stable cell line by using GRP94-specific shRNA in the aggressive CRC cell lines, HCT 116 and DLD-1. Knockdown efficiency was confirmed by western blotting. As shown in Fig. 1a, GRP94 expression was decreased in GRP94-KD by 64.4% for HCT 116 and 81.7% for DLD-1 as compared to scrambled control cells. Furthermore, growth activity of scrambled control and GRP94-KD cells was determined by SRB assay in DLD-1 and HCT 116 cells, respectively. GRP94-KD cells showed reduced cell proliferation as compared to scrambled control cells (Fig. 1b). The results showed that silencing GRP94 suppressed the proliferation of CRC.

### Silencing GRP94 reduces cancer progression in xenograft model

To determine whether GRP94 is required for cancer progression of CRC *in vivo*, the xenograft models were established by implanting scrambled control and GRP94-KD DLD-1 cells into nude mice. The size of tumor was measured twice a week. As presented in Fig. 1c, the size of tumor was increased over time in both scrambled control and GRP94-KD. The volume and weight of scrambled control tumor were markedly larger than those of GRP94-KD (Fig. 1c, d). However, the body weight of the mice was not affected by GRP94 knockdown (data not shown). After sacrificing the mice, the tumors were excised and sectioned for immunohistochemistry analysis. Significantly higher GRP94 signals were detected in the scrambled control as compared to GRP94-KD tumor (Fig. 1e). Therefore, our data indicated that silencing GRP94 may inhibit the tumor growth both *in vitro* and *in vivo*.

### Silencing GRP94 inhibits the metastatic and invasive abilities in CRC cells

**Figure 2:**
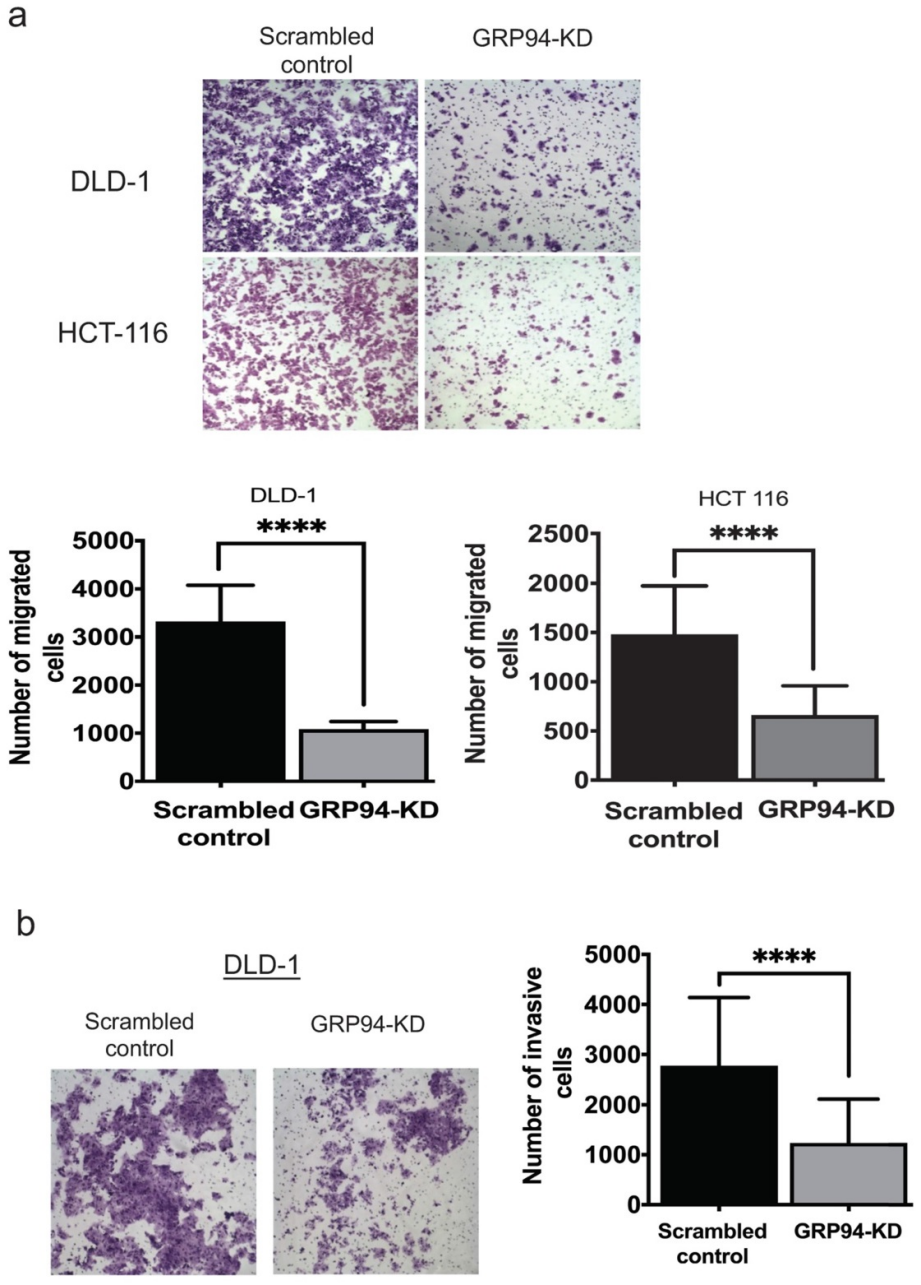
** Knockdown of GRP94 suppresses the migration and invasion ability.** (a) The migratory ability was determined by transwell migration assay. Silencing GRP94 reduced migratory ability in DLD-1 and HCT 116 cells (n=3). (b) Invasive ability was evaluated by transwell invasion assay and was inhibited after silencing GRP94 in DLD-1 cells (n=3). All experiments were repeated at least three times* and* calculated as mean±SD. (****P < 0.0001)

To assess whether GRP94 plays a role in cancer metastasis, transwell migration and invasion assays were used to assess migratory and invasive ability of CRC cells. The results of transwell migration assay showed that GRP94-KD DLD-1 and HCT 116 cells had decreased migratory ability as compared to scrambled control cells (Fig. 2a). Invasive ability was tested in scrambled control and GRP94-KD DLD-1 cells by transwell invasion assay. GRP94-KD cells had significantly fewer invasive cells than scrambled control (Fig. 2b). The results suggest that silencing the expression of GRP94 may suppress the metastatic and invasive abilities in CRC cells.

### Silencing GRP94 reverses epithelial-mesenchymal transition in CRC cells

**Figure 3:**
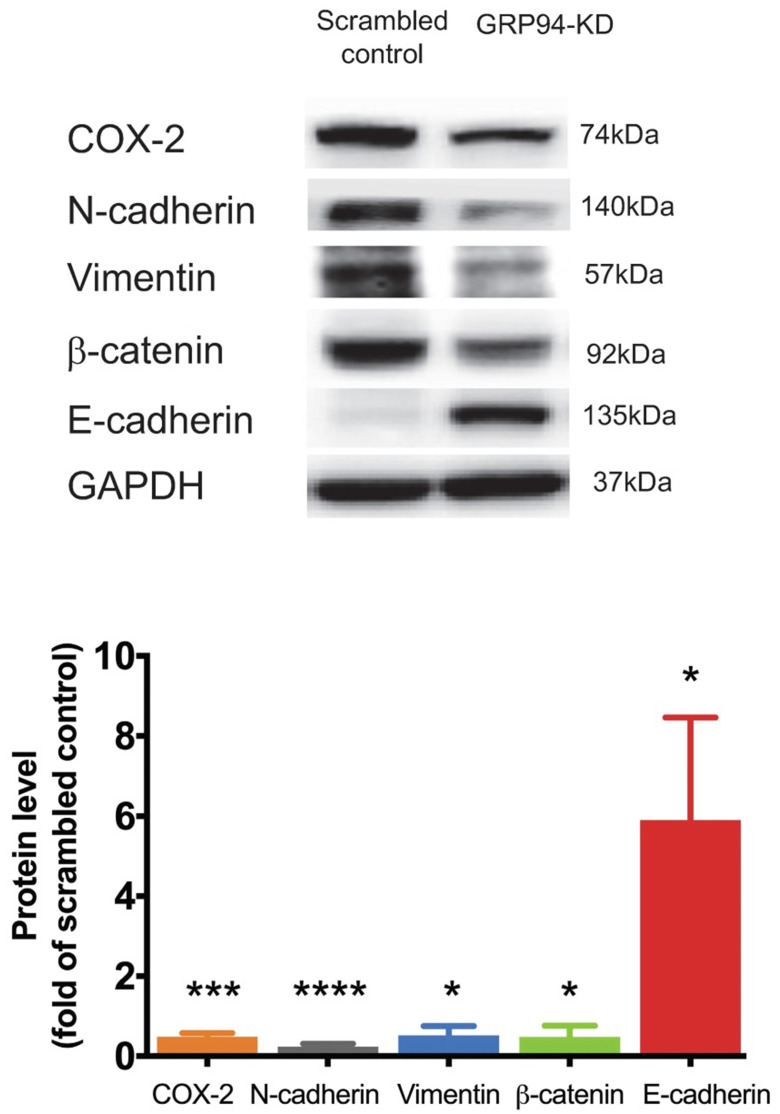
** Silencing GRP94 influences expression of EMT markers.** Level of EMT markers was detected by western blot. Result shows that increased E-cadherin and decreased N-cadherin, COX-2, β-catenin, and vimentin protein levels in GRP94-KD DLD-1 cells (n=3). Data represents mean ±SD of three independent experiments. (*p<0.05, ***p<0.001, ****p<0.0001)

We further analyzed expression levels of mesenchymal markers including Cox-2, N-cadherin, vimentin and β-catenin, and of the epithelial marker including E-cadherin in scrambled control and GRP94-KD DLD-1 cells by Western blotting. The protein levels of Cox-2, N-cadherin, vimentin and β-catenin were decreased by approximately 2-fold in GRP94-KD cells as compared to scrambled control cells. As expected, E-cadherin expression level was 6-fold higher in GRP94-KD cells than that of scrambled control cells (Fig. 3). The results indicate that GRP94-KD might reverse the epithelial-mesenchymal transition (EMT) in CRC cells.

### Knockdown of GRP94 suppresses ETV1 level and MAPK pathway activation

**Figure 4:**
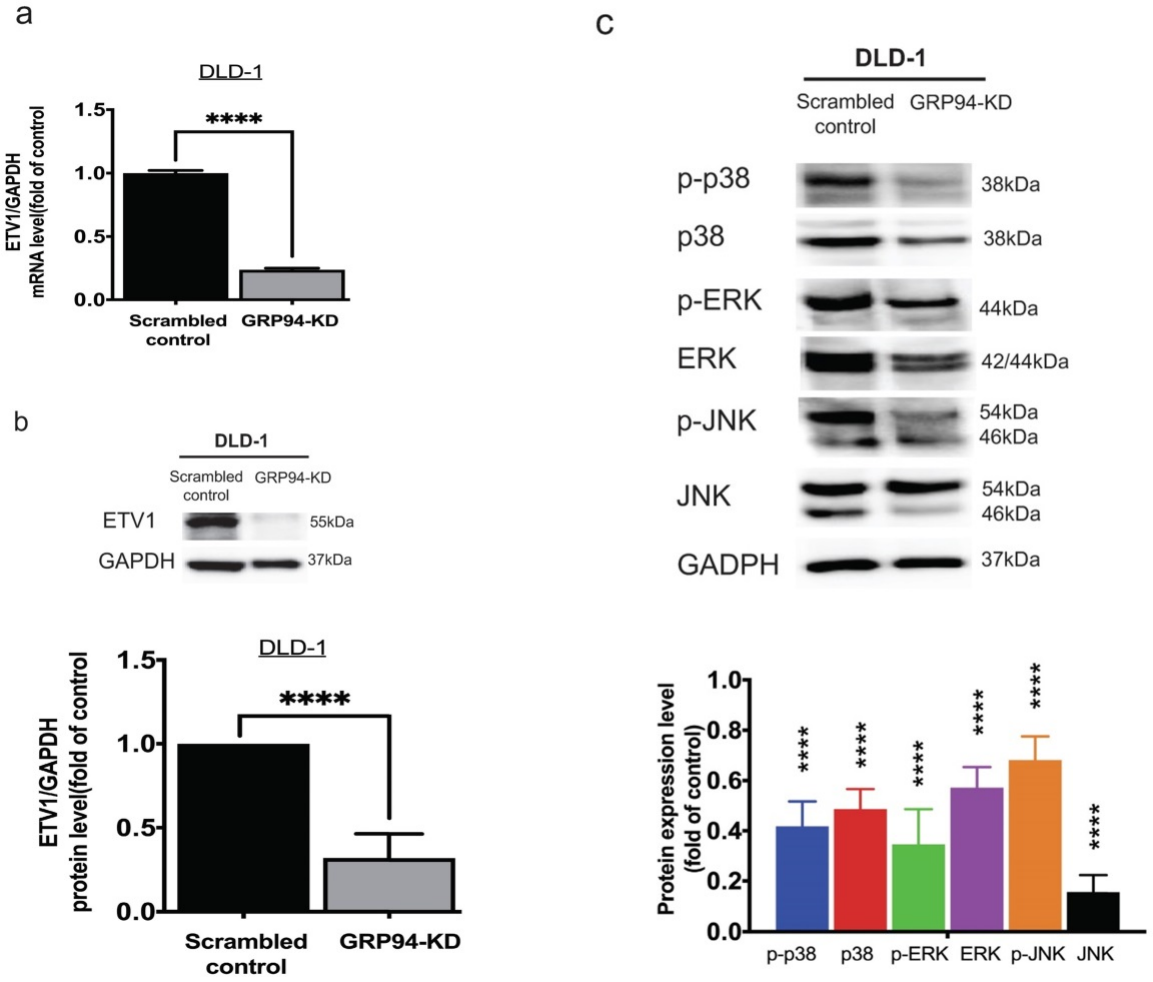
** Knockdown of GRP94 reduced the expression of ETV1 and MAPK pathway proteins in CRC cells.** (a) ETV1 expression level in GRP94-KD DLD-1 cells was detected by QPCR and (b) Western blotting (n=3). (c) Silencing GRP94 caused decreased level of p38, p-p38, ERK, p-ERK, JNK, and p-JNK in MAPK pathway (n=3). All experiments were repeated at least three times and calculated as mean±SD. (****p<0.0001)

To clarify the relationship between GRP94 and ETV1, we evaluated the expression level of ETV1 in scrambled control and GRP94-KD DLD-1 cells by QPCR and Western blot. The mRNA level of ETV1 in GRP94-KD cell was decreased by 80% (Fig. 4a) and the protein expression of ETV1 was reduced by 68%, respectively (Fig. 4b), suggesting that GRP94-KD may suppress ETV1 both at transcriptional and translational level. Previous study showed that inhibitor of GRP94 suppressed the HER2 positive triple negative breast cancers by downregulating the Ras/Raf/MARK pathway 17. Thus, we evaluated the expression of p38, p-p38, ERK, p-ERK, JNK, and p-JNK in the MAPK pathway axis by Western blotting. Expression levels of all the proteins tested in GRP94-KD cells were decreased (Fig. 4c), indicating that silencing GRP94 may inhibit MAPK pathway activation.

### GRP94 mediates cell proliferation and metastasis via ETV1

**Figure 5:**
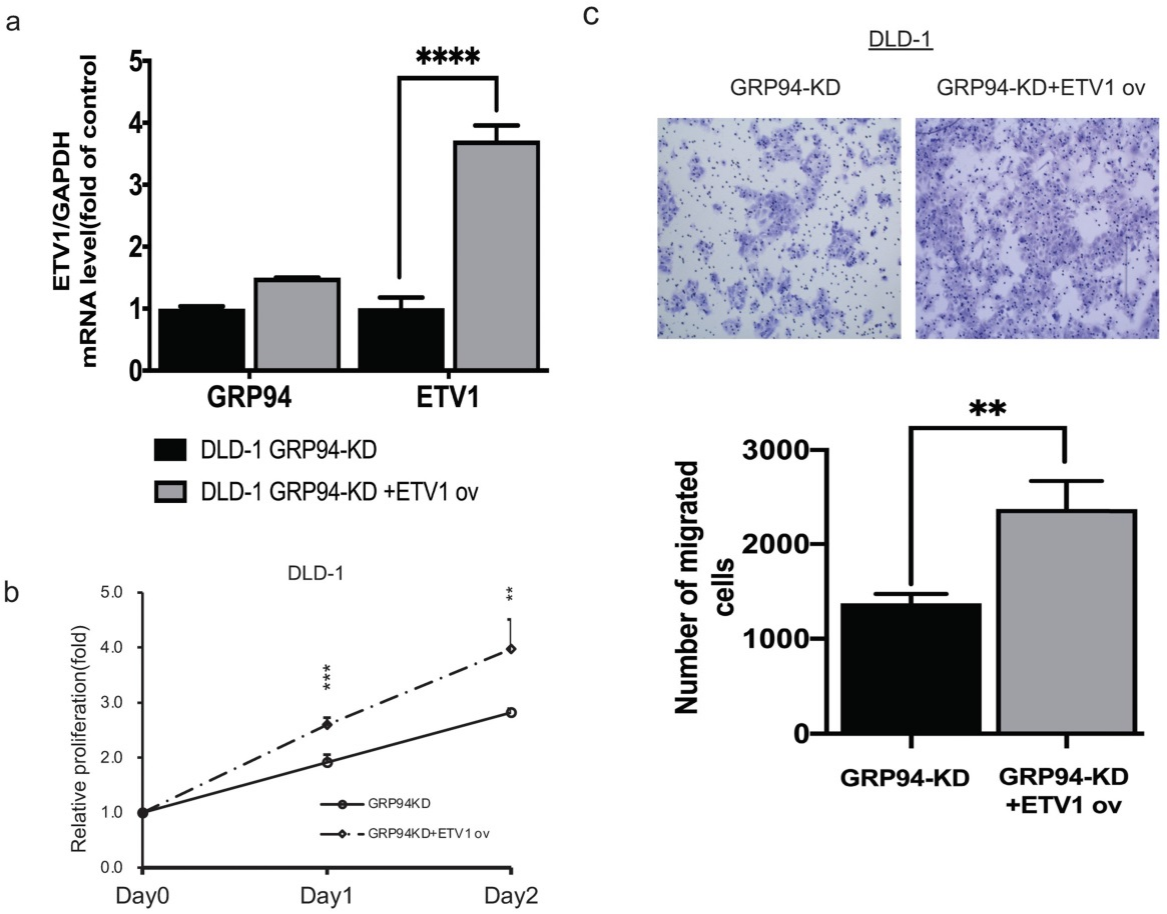
** Overexpression of ETV1 reverses the effect of GRP94 on cell proliferation and migration. (**a) Expression level of ETV1 was determined by qPCR(n=3). (b) SRB assay was performed to evaluate cell proliferation (n=3). Cell growth was increased after overexpressing ETV1 in GRP94-KD cells (c) Cell migration was evaluated by transwell migration assay and overexpression of ETV1 reversed the effect of GRP94 (n=3). (**p<0.01, ***p<0.001, ****p<0.0001)

To confirm whether GRP94 promotes cell proliferation and migration via ETV1, we transiently overexpressed ETV1 expression level in GRP94-KD cells by using electroporation. We checked ETV1 expression by QPCR and found 4-fold increase in GRP94-KD cells (Fig. 5a). After overexpressing ETV1 in GRP94-KD cells, we determined proliferation by SRB and migration ability by transwell migration assays. These results showed that the overexpression of ETV1 reversed the effect of GRP94 on proliferation (Fig. 5b) and migration (Fig. 5c) of GRP94-KD cells.

### Crosstalk between GRP94 and ETV-1 may modulate MAPK pathways

**Figure 6:**
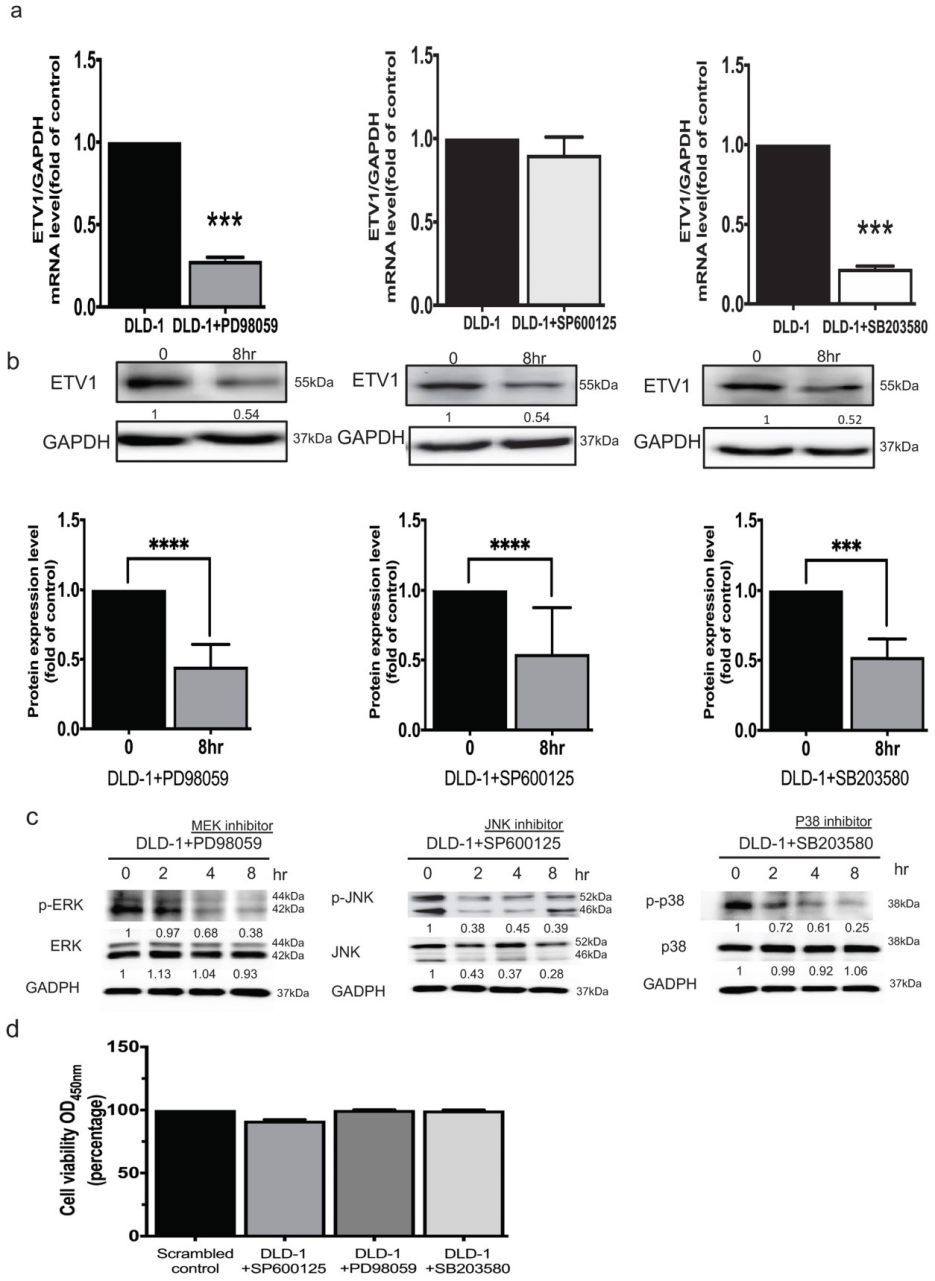
** GRP94 regulates ETV1 expression through MAPK pathway.** (a)The mRNA level of ETV1 was detected by QPCR and was dramatically decreased after treating p38 and ERK inhibitor in DLD-1 cells, while the level of ETV1 was remained unchanged after treatment of JNK inhibitor (n=3). (b) The protein level of ETV1 was determined by western blot and was decreased after treatment of p38(3μM), ERK (50μM), and JNK (50μM) inhibitors (n=3). (c) Protein levels of p38/p-p38, JNK/pJNK, and ERK/pERK were significantly decreased after treatment of p38, JNK, and ERK inhibitors by western blot (n=3). (**p<0.01, ***p<0.001. ****p<0.0001) (d) Cytotoxicity of the inhibitors were determined by CCK8 assay after treating the cells with p38(3μM), ERK (50μM), and JNK (50μM) inhibitors (n=3).

It was reported that ETV1 is a target of the MEK/ERK/Ras/Raf signaling pathway 16. To unravel whether ETV1 is modulated by members of MAPK signal pathways, three specific inhibitors, pd98059 as an ERK inhibitor, sp600125 as a JNK inhibitor, and sb203580 as an Akt/p38 inhibitor, were used. QPCR analysis showed that treating the cells with ERK and Akt/p38 respective inhibitors significantly affected the transcriptional and translational level of ETV1 while JNK inhibitor only affected the translational level (Fig. 6a, b). These data indicated that crosstalk between GRP94 and ETV1 may modulate MAPK pathways. After QPCR analysis, we examined the effect of the inhibitors on the phosphorylation of Akt/p38, ERK, and JNK by western blot. The data showed that all three phosphorylated MAPK signals, p-ERK, p-JNK, and p-p38, were dramatically down-regulated by the specific inhibitors in a time dependent manner (Fig. 6c). Then, we checked the cell viability after treating the cells with same dose of those inhibitors for 8 hours by CCK8 assay, indicating that the inhibitors did not affect the cell viability (Fig. 6d).

## Discussion

GRPs are stress-responsive proteins located mainly in the mitochondria and ER. Different GRPs directed therapeutic compounds are being developed 33, 34. Our previous study found that GRP94-KD played a role in chemoresistance of squamous cells of cervical cancer and esophageal cancer 19, 28. The present study reported the regulatory relationship among GRP94, MAPK, and ETV1 axis.

Screening, early diagnosis, and treatment are the most important issues in the management of CRC patients 35. For treatment, it is much more complex and complicated in advanced, recurrent, or metastatic CRC. Therefore, specific biomarkers for prognostic or therapeutic prediction are urgently needed.

We inspected the impact of GRP94 knockdown on proliferation, invasion, and epithelial-mesenchymal transformation (EMT) in CRC cells. The protein levels of epithelial and mesenchymal markers were evaluated by western blotting in GRP94-KD DLD-1 and scrambled control cells. It showed that silencing GRP94 reversed the EMT in CRC cells and down-regulated Cyclooxygenase-2 (Cox-2), an enzyme that mediates bioconversion of the arachidonic acid into inflammatory prostaglandins and plays an important role in carcinogenesis 36, 37. Cox-2 also contributes to cancer resistance through modulating MDR-1 38. Our data showed that knockdown of GRP94 decreased COX-2 expression and prevent CRC cells from EMT.

ETV1 level of the tyrosine-protein kinase Kit (cKIT) positive interstitial cells has been demonstrated as a survival factor for human gastrointestinal stromal tumors (GIST) 39. It indicated that ETV1 played a role in mesenchymal differentiation and functioned as an identity of stromal cells 40. Previous pancreatic cancer model demonstrated that ETV1 induced invasion, EMT *in vitro*, and promoted stromal expansion and metastasis *in vivo* 23. Both mRNA and protein levels of ETV1 were reduced after silencing GRP94 accompanied by decreasing MAPK signals. To confirm ETV1 as a downstream target for GRP94, we overexpressed ETV1 in GRP94-KD cells and determined proliferation and migration ability. Our results showed that ETV1 overexpression reversed the effect of GRP94 on cell proliferation and migration (Fig.5).

ETV1 can be induced by multiple upstream signaling such as Ras-signaling and TGF-β signaling pathways which are well known for EMT. ETV1 is involved in oncogenesis and is a target of the Ras/Raf/MEK/ERK signaling cascade while the activated ERK-1 is capable of phosphorylating ETV1 16. In our study, several transduction signals of mitogen-activated protein kinase (MAPK) pathways were tested in scrambled control and GRP94-KD DLD-1 cells by western blot. To study the underlying mechanism of GRP94 in CRC, we further treated the DLD-1 cells with ERK, JNK and Akt/p38 specific kinase inhibitors, pd98059, sp600125, and sb203580 respectively and checked ETV1 expression. ERK and Akt/p38 specific inhibitors caused the decreased expression level of ETV1 at both translational and transcriptional level whereas JNK inhibitor only decreased transcriptional level of ETV1 (Fig. 6). We also checked cytotoxicity of inhibitors by treating DLD-1 cells with inhibitors for 8 hours by CCK8 assay. The results showed that all the inhibitors did not affect the cell viability (Fig. 6d).

**Figure 7:**
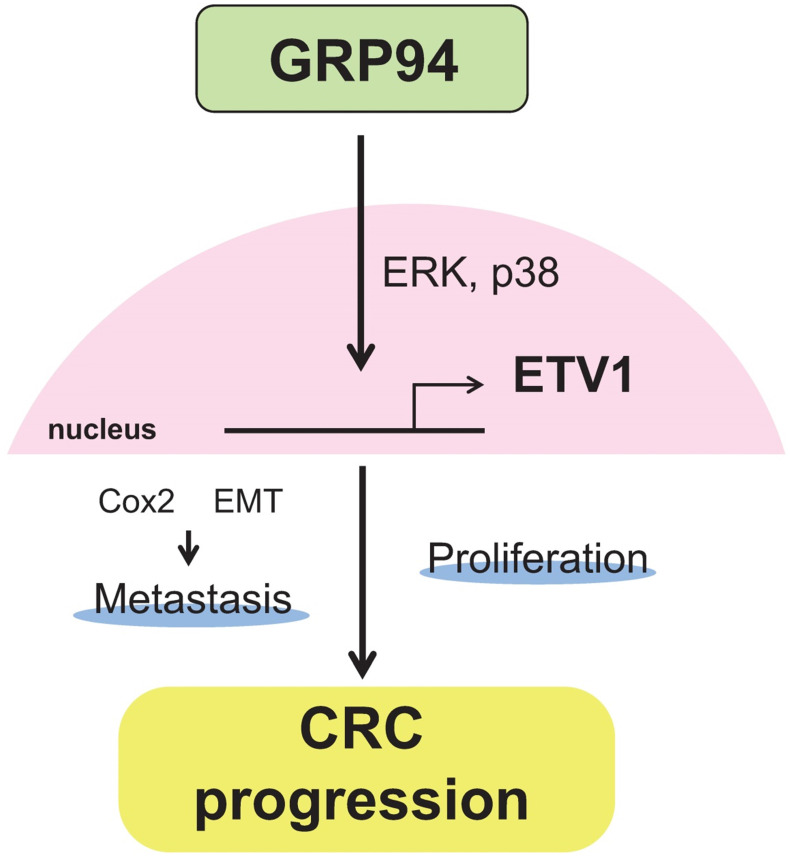
**Schematic diagram illustrating the molecular mechanism of GRP94 involved in colorectal cancer progression.** GRP94 mediates progression of CRC by increasing ETV1 expressions through ERK and p38 and then inducing proliferation and metastasis by enhancing COX2 and EMT.

In summary, our results showed that silencing GRP94 caused reduced tumor progression, invasion, metastasis, and EMT reverse, and down-regulated the expression of ETV1 through inhibition of MAPKs which play a pivotal role in cell proliferation and differentiation (Fig. 7). Additional studies for GRP94/ETV1 may help to clarify the complex crosstalk between tumor cells and stroma in colorectal cancer.
